# Pamidronate Treatment of Osteonecrosis of the Hip in Young Male

**DOI:** 10.2174/1874312901812010125

**Published:** 2018-08-29

**Authors:** A. Lo Gullo, R. Talotta, M. Atteritano

**Affiliations:** Department of Clinical and Experimental Medicine, University of Messina, 98124 Messina, Italy

**Keywords:** Aseptic osteonecrosis, CRP, MRI, Pamidronate, Hip, Dysmotility

## Abstract

**Background:**

Aseptic osteonecrosis of the hip is a clinical entity in which the necrotic process of the bone leads to pain and progressive disability.

**Objective:**

Pamidronate seems to reduce drastically the activation of the osteoclasts so that it can be useful only in the early stage of the disease, delaying the time of bone collapsing.

**Method:**

A 27-years-old male was treated with pamidronate for three consecutive days every four weeks.

**Results:**

After three months the patient came back at control showing a marked improvement in clinical condition, referred full recover from pain and dysmotility with improvement of the quality of life, which was confirmed by the result of MRI he had for control.

**Conclusion:**

We reported a case of aseptic osteonecrosis of the hip which was successfully treated pamidronate at dosage of 45 mg.

## 1 INTRODUCTION

**Fig. (1):**
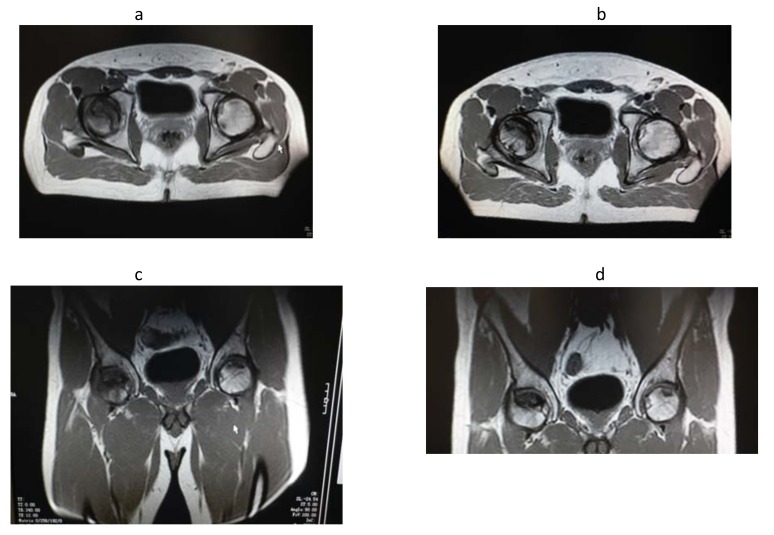
**a** – **c**= MRI images performed at the onset of the disease. **b** – **d**= MRI images performed after three month since start of the treatment with intravenous pamidronate. Reduction of the perifocal oedema and of the dimensions of the two foci of the head of the left femur (blue arrows). The necrotic area present in the head of the right femur was unchanged (orange arrow).

A 27-years-old male attended our clinic with pain and dysmotility at the right hip from 4 months. Six months earlier, he underwent a cardiosurgery intervention due to mitral valvulopathy. He had a history of corticosteroid intake (prednisolone 5 mg/d for 1 month, for cumulative doses of 150 mg) for cutaneous allergic rash assumed in association with vancomycin administration 1-year earlier. He did not refer any alcohol abuse neither previous trauma of the hip. At admission, routine blood exams showed normal values, only C-Reactive Protein (CRP) was mildly increased (1.80 mg/dl; range of normality 0.00-0.50 mg/dl). Magnetic Resonance Imaging (MRI) of the hips performed showed a wide area of hypointensity in T1 sequences, with a diameter of 4x2 cm, at the anterior- superior edge of the right femoral head. A marked bone marrow oedema of the neck of the femur and concomitant joint effusion were detected. In the contralateral femur two small foci of necrosis were also appreciated. We had planned intravenous disodic pamidronate, at dosage of 45 mg for three consecutive days every four weeks, but he received only two of the previewed three infusions because of the development of thromboflebitis of the basilic vein and part of the cefalic and cubital veins of the left forearm, so he was treated with heparin and antibiotics. During the infusion the patient experimented fever (body temperature within 38°C) that was controlled by the administration of paracetamol. The patient was discharged after a further short period of monitoring. After three months, the patient regained control showing a marked improvement in clinical condition referred full recovery from pain and dysmotility with improvement in the quality of life which was confirmed by the result of MRI. (Fig. **1** and Table **1**).

## 2 DISCUSSION

The natural history of aseptic osteonecrosis of the hip, in general, is thought to be one of progressive diseases if no intervention is undertaken. Untreated osteonecrosis of the femoral head is believed to carry a poor prognosis [1-7]. An emphasis has been placed on earlier intervention as it has been associated with an improved outcome [8]. During the first stage of the process, when the activity of the osteoclasts is higher, the use of pamidronate shows some benefits in stopping the course of the disease and in ameliorating the symptoms and the quality of life of the patients [9]. Our case suggests new evidences of the successful use of intravenous pamidronate for the treatment of aseptic osteonecrosis in a young male patient. We still don’t know the future course of the disease, as we assessed the therapeutic vantages after three months of therapy. Besides, MRI showed an improvement of perilesional oedema and the diameter of the two smallest areas of resorption and there was no increase in the dimension of the largest focus on the controlateral hip after three months since the end of the treatment with pamidronate. The use of pamidronate in this case is supported by its role in preventing the activation of the osteoclasts and the death of the osteoblasts, but recently other important functions have been attributed to this drug. Pamidronate can stop the cascade of cytokine activation by inhibiting the secretion of IL-1, IL-6 or TNF-beta from the macrophages, which are induced on the contrary by apoptosis. It therefore can antagonize the development of chronic inflammation and fibrosis [10].

## CONCLUSION

In conclusion, we reported a case of aseptic osteonecrosis of the hip which was successfully treated with two infusion of pamidronate at dosage of 45 mg. Our case could contribute to the therapeutic decisional algorithm acting in favour of the use of bisphosponates.

## Figures and Tables

**Table 1:** Causes of aseptic osteonecrosis.

S.No	Causes
1	Trauma
2	Iatrogenis (Use of glucocorticoids, Alcohol, chemotherapy)
3	Hematological (Thalassemia, Thrombophilia, hemophilia, myeloproliferative disorders)
4	Metabolic (Gaucher disease, hypercholesterolemia, pregnancy, chronic renal failure, Hyperparathyroidism, Cushing’s disease, gout/hyperuricemia, diabetes mellitus)
5	Autoimmune disease (systemic lupus erythematosus, rheumatoid arthritis)
6	Gastrointestinal (Chronic pancreatitis, inflammatiry bowel disease)
7	Orthopaedic (congenital hip dislocation)
8	Reduction of the perifocal oedema and of the dimensions of the two foci of the head of the left femur (blue arrows). The necrotic area present in the head of the right femur was unchanged (orange arrow).
